# Global trans-lesional computed tomography-derived fractional flow reserve gradient is associated with clinical outcomes in diabetic patients with non-obstructive coronary artery disease

**DOI:** 10.1186/s12933-023-01901-9

**Published:** 2023-07-26

**Authors:** Zinuan Liu, Yipu Ding, Guanhua Dou, Xi Wang, Dongkai Shan, Bai He, Jing Jing, Tao Li, Yundai Chen, Junjie Yang

**Affiliations:** 1 Medical School of Chinese PLA, Beijing, China; 2 Senior Department of Cardiology, The Sixth Medical Center, Chinese PLA General Hospital, #6 FuCheng Road, Haidian District, Beijing, China; 3 School of Medicine, Nankai University, Tianjin, China; 4 Department of Cardiology, The Second Medical Center & National Clinical Research Center for Geriatric Diseases, Chinese PLA General Hospital, Beijing, China; 5 Department of Radiology, The First Medical Center of PLA General Hospital, Beijing, China

**Keywords:** Atherosclerosis, Coronary computed tomography angiography, Diabetes mellitus, Fractional flow reserve, Risk stratification

## Abstract

**Background:**

Coronary computed tomography angiography (CCTA)-derived fractional flow reserve (CT-FFR) enables physiological assessment and risk stratification, which is of significance in diabetic patients with nonobstructive coronary artery disease (CAD). We aim to evaluate prognostic value of the global trans-lesional CT-FFR gradient (GΔCT-FFR), a novel metric, in patients with diabetes without flow-limiting stenosis.

**Methods:**

Patients with diabetes suspected of having CAD were prospectively enrolled. GΔCT-FFR was calculated as the sum of trans-lesional CT-FFR gradient in all epicardial vessels greater than 2 mm. Patients were stratified into low-gradient without flow-limiting group (CT-FFR > 0.75 and GΔCT-FFR < 0.20), high-gradient without flow-limiting group (CT-FFR > 0.75 and GΔCT-FFR ≥ 0.20), and flow-limiting group (CT-FFR ≤ 0.75). Discriminant ability for major adverse cardiovascular events (MACE) prediction was compared among 4 models [model 1: Framingham risk score; model 2: model 1 + Leiden score; model 3: model 2 + high-risk plaques (HRP); model 4: model 3 + GΔCT-FFR] to determine incremental prognostic value of GΔCT-FFR.

**Results:**

Of 1215 patients (60.1 ± 10.3 years, 53.7% male), 11.3% suffered from MACE after a median follow-up of 57.3 months. GΔCT-FFR (HR: 2.88, 95% CI 1.76–4.70, *P* < 0.001) remained independent risk factors of MACE in multivariable analysis. Compared with the low-gradient without flow-limiting group, the high-gradient without flow-limiting group (HR: 2.86, 95% CI 1.75–4.68, *P* < 0.001) was associated with higher risk of MACE. Among the 4 risk models, model 4, which included GΔCT-FFR, showed the highest C-statistics (C-statistics: 0.75, *P* = 0.002) as well as a significant net reclassification improvement (NRI) beyond model 3 (NRI: 0.605, *P* < 0.001).

**Conclusions:**

In diabetic patients with non-obstructive CAD, GΔCT-FFR was associated with clinical outcomes at 5 year follow-up, which illuminates a novel and feasible approach to improved risk stratification for a global hemodynamic assessment of coronary artery in diabetic patients.

**Supplementary Information:**

The online version contains supplementary material available at 10.1186/s12933-023-01901-9.

## Background

Although coronary computed tomography angiography (CCTA) has become the first-line recommendation for evaluating coronary artery disease (CAD), functional information may provide additional benefits beyond anatomic characteristics for lesion identification and risk assessment [1, 2]. CCTA-derived fractional flow reserve (CT-FFR) is an emerging technique based on computational fluid dynamics which allows to assess the hemodynamic significance of coronary artery stenosis [3]. Nevertheless, a discrepancy was revealed between CT-FFR and coronary stenosis severity in patients with negative CT-FFR, who received inadequate treatment due to being considered “negative”, were somewhat prone to adverse events [4, 5].

CT-FFR lesional gradient (ΔCT-FFR) may represent a reliable solution that can improve the discrimination of those underwent early revascularization [6]. In this regard, we hypothesized that the sum of ΔCT-FFR in epicardial vessels might have a stronger and more direct hemodynamic correlation with the global coronary atherosclerotic burden by reflecting the reduction in hyperemic epicardial conductance pressure in the whole coronary artery tree. Diabetes mellitus, in particular, may benefit from such a global evaluation approach in the presence of multi-vessel and diffuse lesion disturbance. To prove this concept, we defined global ΔCT-FFR (GΔCT-FFR) as the sum of the ΔCT-FFR in all epicardial vessels. The present study aimed to investigate whether GΔCT-FFR could identify the diabetic individual at high risk of long-term clinical outcomes even in the absence of hemodynamically significant lesions.

## Methods

### Study population

**Fig. 1:**
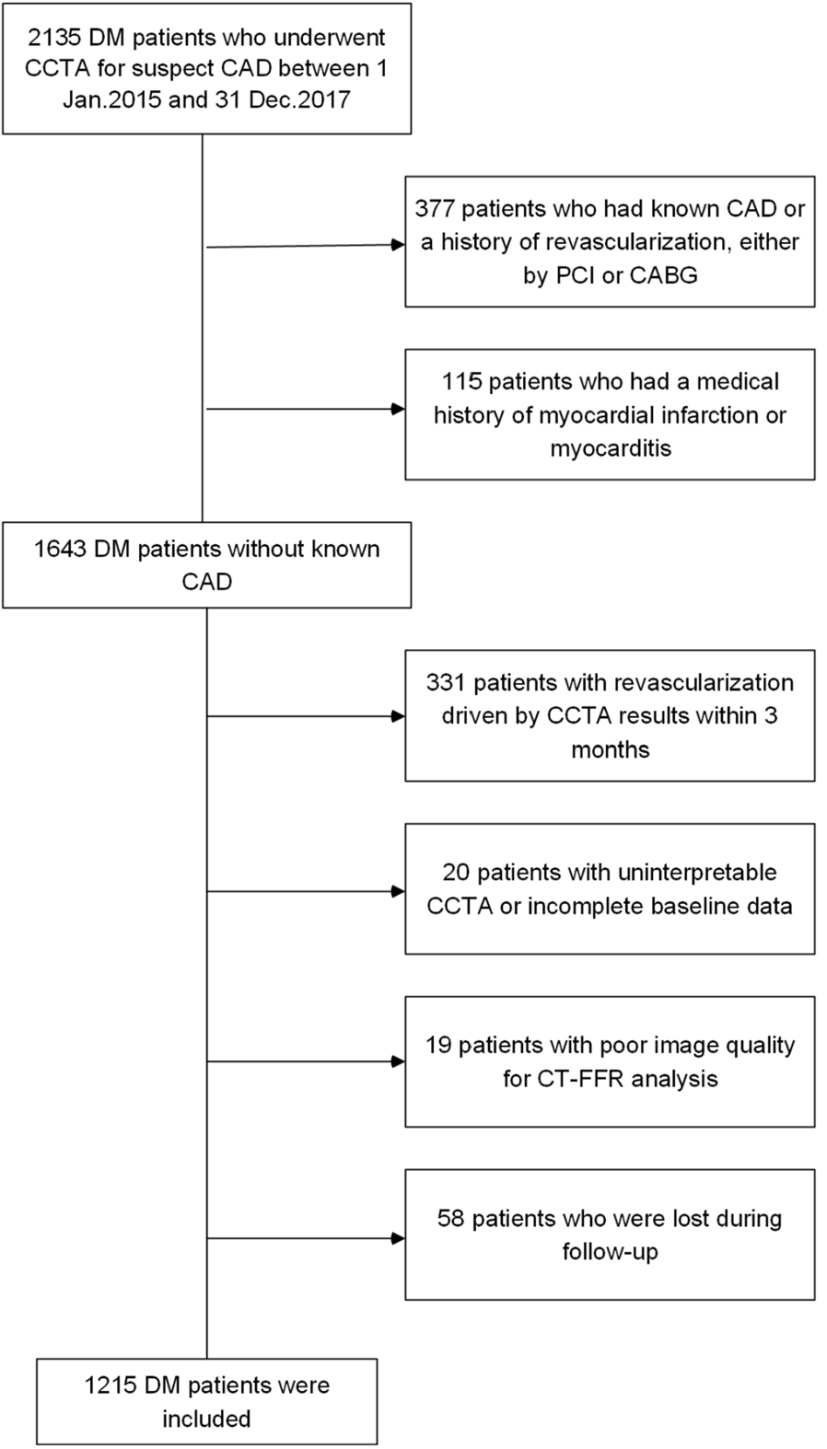
Study flowchart. *DM* diabetes mellitus, *CCTA* coronary computed tomography angiography, *CAD* coronary artery disease, *PCI* percutaneous coronary intervention, *CABG* coronary artery bypass grafting, *CT-FFR* computed tomography–derived fractional flow reserve

This was a prospective, single-site cohort study. It involved patients with type 2 diabetes investigated for clinically suspected CAD using CCTA between January 2015 and December 2017. Of 1643 patients with diabetes without known CAD [history of coronary revascularization (either coronary artery bypass grafting or percutaneous coronary intervention), myocardial infarction, or myocarditis], 331 patients with revascularization based on CCTA results within 3 months, 20 patients with uninterpretable CCTA or incomplete baseline data, 19 patients failed to undergo CT-FFR measurement due to insufficient image quality and 58 patients lost to follow-up were excluded, leaving 1215 patients for further analysis (Fig. 1). All patients have been previously reported [7]. This prior article dealt with the prognostic value of atherosclerotic extent indicated by CCTA-derived risk score system in diabetic patients with nonobstructive CAD whereas in this manuscript we focused on the prognosis of the global trans-lesional CT-FFR gradient (GΔCT-FFR) in patients with diabetes without flow-limiting stenosis. In addition, the follow-up time was extended in the present work.

The study protocol conformed to the ethical guidelines of the 1975 Declaration of Helsinki, and informed consent was obtained for all participants before CCTA or CT-FFR examination. Ethical approval for the study was obtained from the local ethics committee (Approval S2020-255-01).

### Clinical data

The demographical and clinical information were collected through an integrated electronic medical record system. According to the 2019 American Diabetes Association guidelines [8], diabetes was defined as fasting plasma glucose ≥ 7.0 mmol/L, 2-h plasma glucose ≥ 11.1 mmol/L during oral glucose tolerance test, A1C ≥ 6.5% (48 mmol/mol), or a previous diagnosis of diabetes (medical diagnosis or the use of insulin/oral hypoglycemic agents). Hypertension was defined as systolic blood pressure ≥ 140 mm Hg, diastolic blood pressure ≥ 90 mm Hg, or a documented history of high blood pressure (including treatment with antihypertensive medication) [9]. Hyperlipidemia was defined as serum total cholesterol ≥ 230 mg/dL, serum triglycerides ≥ 200 mg/dL or a documented history of dyslipidemia (including the use of lipid-lowering medication) [10]. Smoking was defined as current or previous smoking within the last 3 months before CCTA. Family history was considered if CAD was presented in first-degree family members at the age of < 55 years in men or < 65 years in women [11].

### CCTA acquisition and interpretation

CCTA was performed on a dual-source CT scanner (Somatom Definition Flash CT, Siemens Medical Solutions, Forchheim, Germany) following local practice and international guidelines. Image data were transferred to a dedicated workstation (Syngo. via VB10B, Siemens Healthcare, Forchheim, Germany) for further analysis. CCTA interpretation was conducted by two experienced cardiologists blind to clinical outcomes based on a 17-segment coronary tree model. In the case of disagreement, a third professional expert was consulted.

For each segment, the coronary lesion severity was reported as percent diameter stenosis by visual assessment and stratified using the Coronary Artery Disease – Reporting and Data System (CAD-RADS) [12] as CAD-RADS 0 (0% stenosis), CAD-RADS 1 (1−24% stenosis), CAD-RADS 2 (25−49% stenosis), CAD-RADS 3 (50-69% stenosis), CAD-RADS 4 A (70**−**99% stenosis in 1−2 vessels), CAD-RADS 4B (70-99% stenosis in 3 vessels or ≥ 50% in the left main vessel), and CAD-RADS 5 (100% stenosis or total occlusion). High-risk plaque (HRP) was also detected visually as the coexistence of at least 2 vulnerable characteristics [13], including spotty calcification (calcification < 3 mm in length and comprising < 90° of the vessel circumference) [14], positive remodeling [remodeling index (the ratio of the stenosis vessel diameter to the standard reference vessel diameter) > 1.1] [15], low-density plaques (attenuation < 30 Hounsfield) [15], and napkin-ring sign (a low attenuation central core surrounded by a ring-like peripheral higher attenuation) [16]. The segment involvement score (SIS), segment stenosis score (SSS) [17], and Leiden score [10] were calculated to quantify the atherosclerosis burden and further subdivided into < 3 and ≥ 3 for SIS, < 5 and ≥ 5 for SSS, < 5, 5–20 and > 20 for Leiden. Scores and weights were calculated in Leiden score, including coronary anatomical dominance, location of stenosis, stenosis severity and plaque composition. While only the segment involved or stenosis severity were considered in SIS or SSS.

### CT-FFR examination and measurement

Deep-learning-based CT-FFR was performed on a dedicated, commercially available software (DEEPVESSEL FFR, KeyaMed NA inc. Seattle, USA) by experts blind to the clinical outcomes without affecting clinical management. This software has been approved by the US Food and Drug Administration for 510(k) clearance. Briefly, the modified 3D U-Net-like model was employed to generate and extract the coronary artery tree and the centerlines. Then, the novel path-based deep learning model was used to predict the simulated FFR values at any point on the coronary artery tree model [18, 19].

CT-FFR evaluations were performed for patients with CAD-RADS 0–4. Lesion-specific CT-FFR was measured at 10–20 mm distal to the end of the stenosis [20]. Per-patient CT-FFR was defined as the lowest lesion-specific CT-FFR value detected from the lesion of the whole coronary tree. Considering lower CT-FFR values than measured invasive FFR with a bias ranging between 0.03 and 0.05 and the highest agreement when CT-FFR was less than or equal to 0.75 [20], we rendered CT-FFR ≤ 0.75 as flow-limiting coronary stenosis (obstructive), and CT-FFR > 0.75 as non-flow limiting stenosis (non-obstructive), which was also in line with the previous study [21]. ΔCT-FFR represented the change in CT-FFR across the lesion and was measured as the difference in CT-FFR approximately 10–20 mm proximal and distal to the stenosis as follows:

$$\Delta\mathrm{CT}-\mathrm{FFR}=\mathrm{proximal\,CT}-\mathrm{FFR}\,-\,\mathrm{distal\,CT}-\mathrm{FFR}$$

GΔCT-FFR comprised the sum of the ΔCT-FFR in the epicardial vessels greater than 2 mm, considered as a hemodynamic correlative of the global coronary atherosclerotic burden, and was calculated as follows:

$$G\Delta\mathrm{CT}-\mathrm{FFR}=\Sigma\Delta\mathrm{CT}-\mathrm{FFR}(\mathrm{coronary}\;\mathrm{arteries}\;\mathrm{larger}\;\mathrm{than}\;2\mathrm{mm})$$

**Fig. 2:**
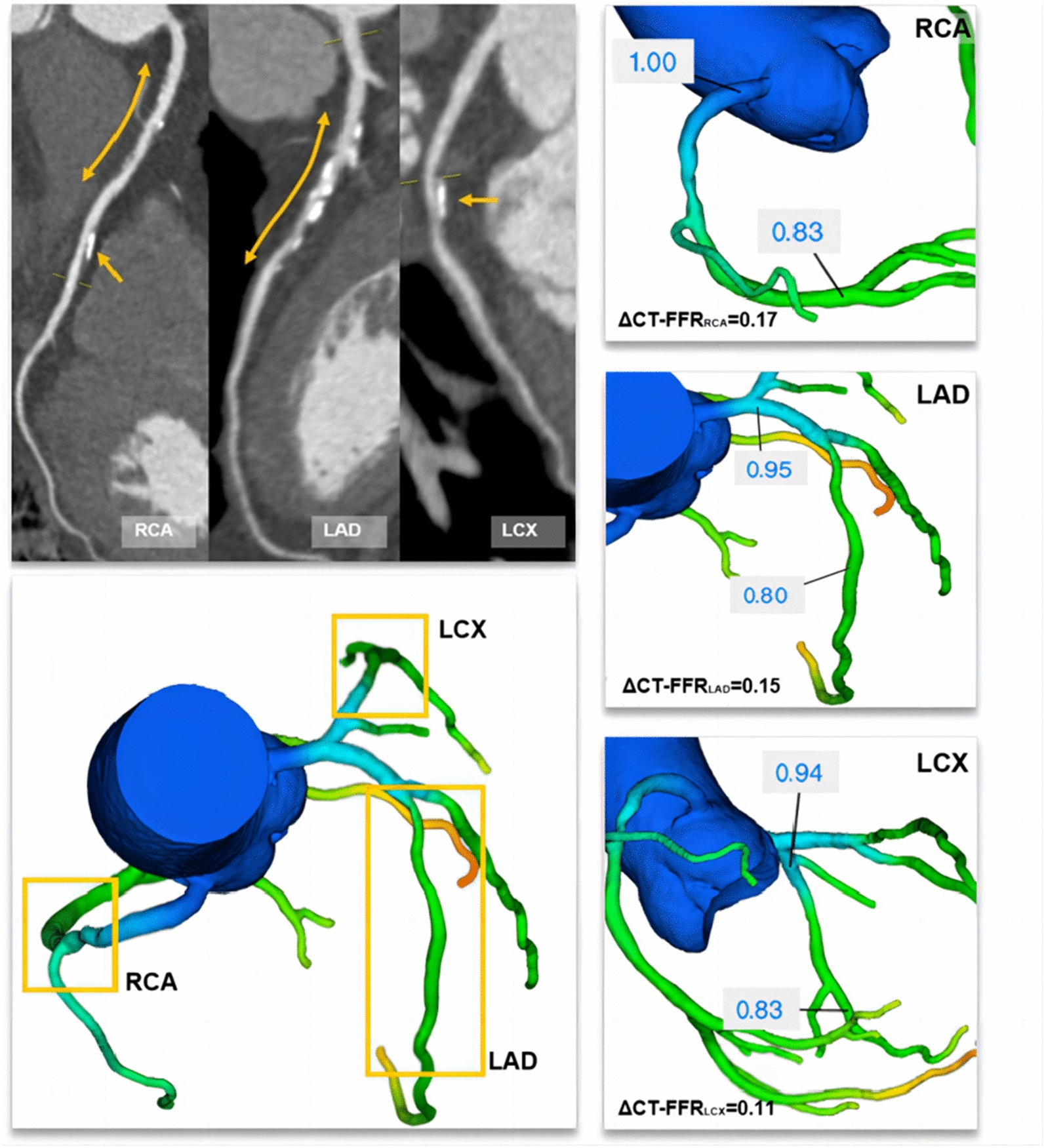
A representative case of obstructive coronary artery disease with negative CT-FFR but a high global ΔCT-FFR. *CT-FFR* coronary computed tomography angiography−derived fractional flow reserve, *ΔCT-FFR* CT-FFR lesional gradient, *LAD* left anterior descending artery, *LCX* left circumflex artery, *RCA* right coronary artery. A 60-year-old man with a history of diabetes mellitus underwent a CCTA examination for suspected CAD. CCTA showed 30% stenosis (orange arrow) in the proximal and middle RCA, 70% stenosis in the proximal LAD and 30% stenosis in the proximal LCX. The CT-FFR value was 0.83, 0.80, and 0.83 for the RCA, LAD, and LCX systems, respectively. The corresponding ΔCT-FFR value was 0.17, 0.15, and 0.11, respectively. Thus, the global ΔCT-FFR was calculated as the sum of the ΔCT-FFR of 0.43

**Fig. 3:**
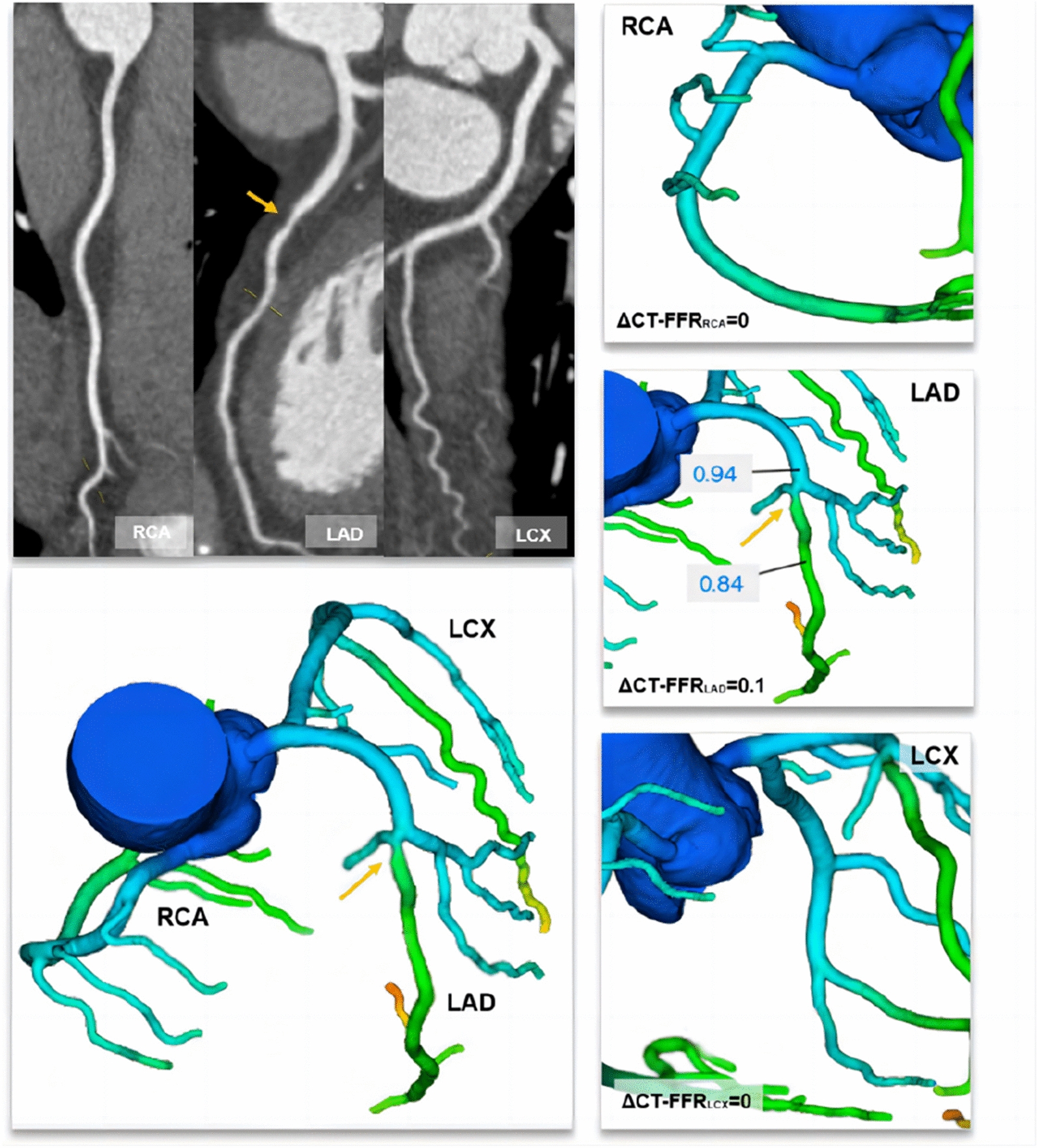
A representative case of nonobstructive coronary artery disease with negative CT-FFR and a low global ΔCT-FFR. *CT-FFR* coronary computed tomography angiography−derived fractional flow reserve, *ΔCT-FFR* CT-FFR lesional gradient, *LAD* left anterior descending artery, *LCX* left circumflex artery, *RCA* right coronary artery. A 55-year-old man with a history of diabetes mellitus underwent a CCTA examination for suspected CAD. CCTA showed 40% stenosis (orange arrow) in the proximal LAD and no significant stenosis in the RCA and LCX. The CT-FFR value was 0.90, 0.84, and 0.92 for the RCA, LAD, and LCX systems, respectively. ΔCT-FFR was 0.10 for LAD and 0 for RCA and LCX because no ischemic plaque was found. Thus, the global ΔCT-FFR was calculated as the sum of the ΔCT-FFR of 0.10

Similar to invasive FFR, default CT-FFR values of 0.5 was assigned to totally occluded arteries, as previously described [22], thus, ΔCT-FFR was also 0.5 as default. Considering the diffuse lesions and severe stenosis in patients with occluded arteries, GΔCT-FFR was 1.5 as default (i.e., the extreme case of occlusion of all 3 major arteries). The calculation examples of ΔCT-FFR and GΔCT-FFR are presented in Figs. 2 and 3.

### Outcomes

The follow-up data were obtained through the review of electronic medical records or telephonic interviews by trained personnel at regular intervals at least 90 days after the CCTA examination for all patients. The primary outcome of the present study was major adverse cardiovascular events (MACE), including cardiovascular death, nonfatal myocardial infarction, and unstable angina requiring hospitalization. Major adverse cardiovascular and cerebrovascular events (MACCE) were determined as the secondary outcome for sensitivity analyses, including MACE and ischemic stroke. Two independent cardiologists adjudicated these events blind to treatment allocation, CCTA and CT-FFR results.

### Statistical analysis

Continuous variables were presented as mean ± standard deviation or median with interquartile range, whereas categorical variables were presented as frequencies or percentages. Normally distributed variables were tested using the Student *t-*test, whereas the Mann–Whitney *U* test was used for skewness distributed variables. The chi-square test was performed for categorical variables. Hemodynamically significant CAD was defined by the value of CT-FFR ≤ 0.75. For those without flow-limiting stenosis, the patients were further classified by GΔCT-FFR at a cutoff value of 0.20. Kaplan–Meier analysis was used for survival estimation and comparison for subgroups with the log-rank test. Cox proportional hazards regression was used to calculate the hazard ratio (HR) and 95% confidence interval (CI) to ascertain the independent contribution of GΔCT-FFR to MACE. Moreover, to determine the incremental prognostic value of GΔCT-FFR compared with clinical risk factors and coronary anatomic characteristics, the C-statistic and receiver operating characteristic (ROC) curve were performed and compared. A sensitivity analysis was also conducted with MACCE as the outcome to prove the robustness of GΔCT-FFR for risk stratification among patients with non- flow limiting stenosis. A two-tailed *P* value < 0.05 indicated a statistically significant difference. Statistical analysis was performed with SPSS version 26.0 (SPSS, IL, USA) and R version 4.2.1(including the “rms” and “pROC” packages).

## Results

### Patient demographics and baseline characteristics

A total of 1215 participants were included in the final analysis (Fig. 1). At a median follow-up of 57.3 months (interquartile range, 51.1–70.7 months), 137 (11.3%) MACE occurred, including 8 cardiovascular deaths, 31 nonfatal myocardial infarctions, and 98 unstable angina requiring hospitalization, while MACCE was 171 (14.1%) with 35 strokes (one patient had both unstable angina requiring hospitalization and ischemia stroke). The average age was 60.1 years, 53.7% were men, and 68.0% had a CAD-RADS of 1–3, as demonstrated by CCTA. Patients with MACE were at high prevalence of hyperlipidemia (68.6% vs. 51.9%; *P* < 0.001), current smoking (35.0% vs. 25.9%; *P =* 0.02), higher Framingham risk score [19 (17–21.5) vs. 18 (16–20); P = 0.001], HRP (16.8% vs. 3.8%; *P* < 0.001), higher CAD-RADS [3 (2–4) vs. 2(1–3); *P* < 0.001], and multi-segment disease as SIS quantified (SIS ≥ 3: 69.3% vs. 35.3%; *P* < 0.001) compared with those without MACE (Tables 1,2).

**Table 1:** Baseline characteristics

Characteristic	No MACE (*N* = 1078)	MACE (*N* = 137)	*P* value
Age (year)	59.9 ± 10.2	61.4 ± 10.7	0.16
Male	570 (52.9%)	82 (59.9%)	0.12
Body mass index (kg/m²)	26.2 ± 3.6	26.3 ± 3.5	0.33
Cardiac risk factors			
Hypertension	716 (66.4%)	94 (68.6%)	0.61
Hyperlipidemia	560 (51.9%)	94 (68.6%)	< 0.001
Current smoking	279 (25.9%)	48 (35.0%)	0.02
Family history of CAD	262 (24.3%)	33 (24.1%)	0.96
Framingham risk score	18 (16–20)	19 (17–21.5)	0.001
< 10%	8 (0.7%)	0 (0.0%)	0.31
10–20%	836 (77.6%)	90 (65.7%)	0.002
> 20%	234 (21.7%)	47 (34.3%)	0.001
Medication			
Anti-platelet	408 (37.8%)	59 (43.1%)	0.24
Beta blocker	356 (33.0%)	45 (32.8%)	0.97
ACEI/ARB	243 (22.5%)	37 (27.0%)	0.24
Statin	395 (36.6%)	75 (54.7%)	< 0.001
Calcium channel blocker	222 (20.6%)	37 (27.0%)	0.08
Diabetic treatment			
Diet only	219 (20.3%)	23 (16.8%)	0.33
Oral hypoglycemic agent^a^	787 (73.0%)	98 (71.5%)	0.72
Insulin	253 (23.5%)	41 (29.9%)	0.10

Values are mean ± standard deviation, median (interquartile range) or *n* (%)

*CAD* coronary artery disease, *MACE* major adverse cardiovascular events

^a^Oral hypoglycemic agent included those from the biguanide, thiazolidinedione, sulfonylurea, meglitinide classes, α-glucosidase inhibitors, DPP-4 inhibitor and SGLT2 inhibitors

**Table 2:** CCTA findings

Characteristic	No MACE (N = 1078)	MACE (N = 137)	P value
CAD-RADS	2 (1–3)	3 (2–4)	< 0.001
0	184 (17.1%)	5 (3.6%)	< 0.001
1–2	579 (53.7%)	38 (27.7%)	< 0.001
3–4	300 (27.8%)	86 (62.8%)	< 0.001
5	15 (1.4%)	8 (5.8%)	< 0.001
High-risk plaque	41 (3.8%)	23 (16.8%)	< 0.001
Segment involvement score	1 (1–3)	3 (2–6)	< 0.001
< 3	698 (64.7%)	42 (30.7%)	< 0.001
≥ 3	380 (35.3%)	95 (69.3%)	< 0.001
Segment stenosis score	2 (1–4)	6 (3–10)	< 0.001
<5	841 (78.0%)	56 (40.9%)	< 0.001
≥5	237 (22.0%)	81 (59.1%)	< 0.001
Leiden risk score	4.6 (1.2–10.2)	11.2 (5.6–15.7)	< 0.001
< 5	585 (54.3%)	25 (18.2%)	< 0.001
5–20	446 (41.4%)	95 (69.3%)	< 0.001
> 20	47 (4.4%)	17 (12.4%)	< 0.001
Vessels affected			
One-vessel disease	251 (23.3%)	20 (14.6%)	0.02
Two-vessel disease	219 (20.3%)	40 (29.2%)	0.02
Three-vessel disease	211 (19.6%)	57 (41.6%)	< 0.001
CACS			
0	632 (58.6%)	58 (42.3%)	< 0.001
1–100	230 (21.3%)	27 (19.7%)	0.66
100–300	99 (9.2%)	17 (12.4%)	0.23
> 300	117 (10.9%)	35 (25.5%)	< 0.001

Values are n (%) or median (interquartile range)

*CCTA* coronary computed tomography angiography, *MACE* major adverse cardiovascular events, *CAD-RADS* coronary artery disease-reporting and data system, *CACS* coronary artery calcium score

### Hemodynamic metrics

On per-patient level, the mean CT-FFR was 0.86 ± 0.09, with 10.8% (131 of 1215) of patients having a CT-FFR of ≤ 0.75, and the mean GΔCT-FFR was 0.19 ± 0.25. Patients with lower CT-FFR and higher GΔCT-FFR, representing relatively hemodynamic abnormalities, were significantly more likely to have MACE (Table 3).

**Table 3:** Hemodynamic characteristics

Hemodynamic characteristics	No MACE (N = 1078)		MACE (N = 137)	P value
CT-FFR	0.87 ± 0.08		0.79 ± 0.11	< 0.001
CT-FFR ≤ 0.75	89 (8.3%)		42 (30.7%)	< 0.001
GΔCT-FFR	0.17 ± 0.22		0.37 ± 0.36	< 0.001
GΔCT-FFR ≥ 0.20	247 (22.9%)		86 (62.8%)	< 0.001

*CT-FFR* coronary computed tomography angiography−derived fractional flow reserve, *GΔCT-FFR* Global ΔCT-FFR (as the sum of the trans-lesional CT-FFR gradient in all epicardial vessels), *MACE* major adverse cardiovascular events

### Cumulative incidence of MACE

**Fig. 4:**
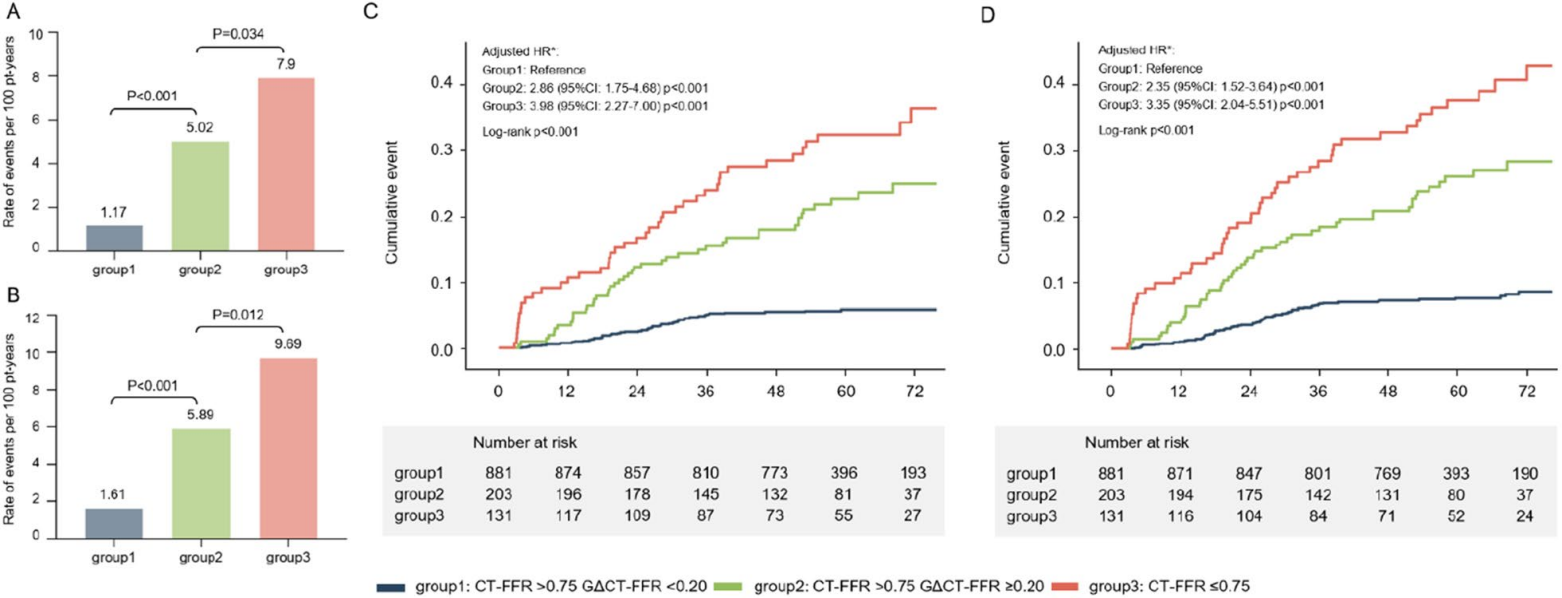
Cumulative incidence of primary **A** and secondary **B** endpoint events for stratified groups based on CT-FFR and global ΔCT-FFR, and Kaplan–Meier survival curves for MACE-free survival of primary **C** and secondary endpoint **D**. *Adjusted for age, sex, hyperlipidemia, smoking, HRP and Leiden score. *HRP* high-risk plaque, *CT-FFR* coronary computed tomography angiography−derived fractional flow reserve, *GΔCT-FFR* Global ΔCT-FFR (the sum of the trans-lesional CT-FFR gradient in all epicardial vessels)

Of the subgroups stratified by CT-FFR and GΔCT-FFR, an incremental trend of event incidence is demonstrated in Fig. 4. The annual rate of MACE was 1.17 events per 100 person-years for the low-gradient without flow-limiting group (CT-FFR > 0.75 and GΔCT-FFR < 0.20), 5.02 events per 100 person-years for the high-gradient without flow-limiting group (CT-FFR > 0.75 and GΔCT-FFR ≥ 0.20), and 7.90 events per 100 person-years for the flow-limiting group (CT-FFR ≤ 0.75) respectively (Fig. 4A). A significant difference in survival rates was found between groups (log-rank P < 0.001) (Fig. 4A and C).

### Incremental prognostic value of GΔCT-FFR in predicting MACE

After adjustment, GΔCT-FFR (HR:2.88, 95% CI 1.76–4.70, *P* < 0.001) was still significantly associated with the increased occurrence of MACE (Table 4). Of the subgroups, an increase in risk was observed for the high-gradient without flow-limiting group (HR: 2.86, 95% CI 1.75–4.68, *P* < 0.001) compared with the low-gradient without flow-limiting group after adjustment (Fig. 4C), while the flow-limiting group was associated with a higher risk of MACE (HR: 3.98, 95% CI 2.27–7.00, *P* < 0.001).

**Table 4:** Multivariate analysis for prediction of outcomes

Characteristic	MACE			MACCE		
	Multivariate HR (95% CI)	Wald *x*²	P value	Multivariate HR (95% CI)	Wald *x*²	P value
Age (year)	1.00 (0.98–1.02)	0.005	0.94	1.00 (0.99–1.02)	0.288	0.59
Male	0.98 (0.67–1.45)	0.007	0.93	1.10 (0.78–1.56)	0.293	0.59
Hyperlipidemia	1.93 (1.34–2.77)	12.413	< 0.001	1.61 (1.18–2.21)	8.800	0.003
Current smoking	1.19 (0.80–1.77)	0.739	0.39	1.06 (0.74–1.52)	0.111	0.74
High-risk plaque	2.67 (1.68–4.24)	17.083	< 0.001	2.35 (1.52–3.64)	14.686	< 0.001
Leiden score	1.43 (0.98–2.08)	3.508	0.06	1.50 (1.07–2.08)	5.676	0.02
CT-FFR ≤ 0.75	1.40 (0.90–2.16)	2.229	0.14	1.43 (0.96–2.13)	3.012	0.08
GΔCT-FFR ≥ 0.20	2.88 (1.76–4.70)	17.734	< 0.001	2.37 (1.53–3.66)	15.041	< 0.001

*CI* confidence interval, *CT-FFR* coronary computed tomography angiography-derived fractional flow reserve, *GΔCT-FFR* global ΔCT-FFR (sum of the trans-lesional CT-FFR gradient in all epicardial vessels), *HR* hazard ratio, *MACE* major adverse cardiovascular events, *MACCE* major adverse cardiovascular and cerebrovascular events

### Risk stratification for GΔCT-FFR in diabetic patients with nonobstructive CAD

**Fig. 5:**
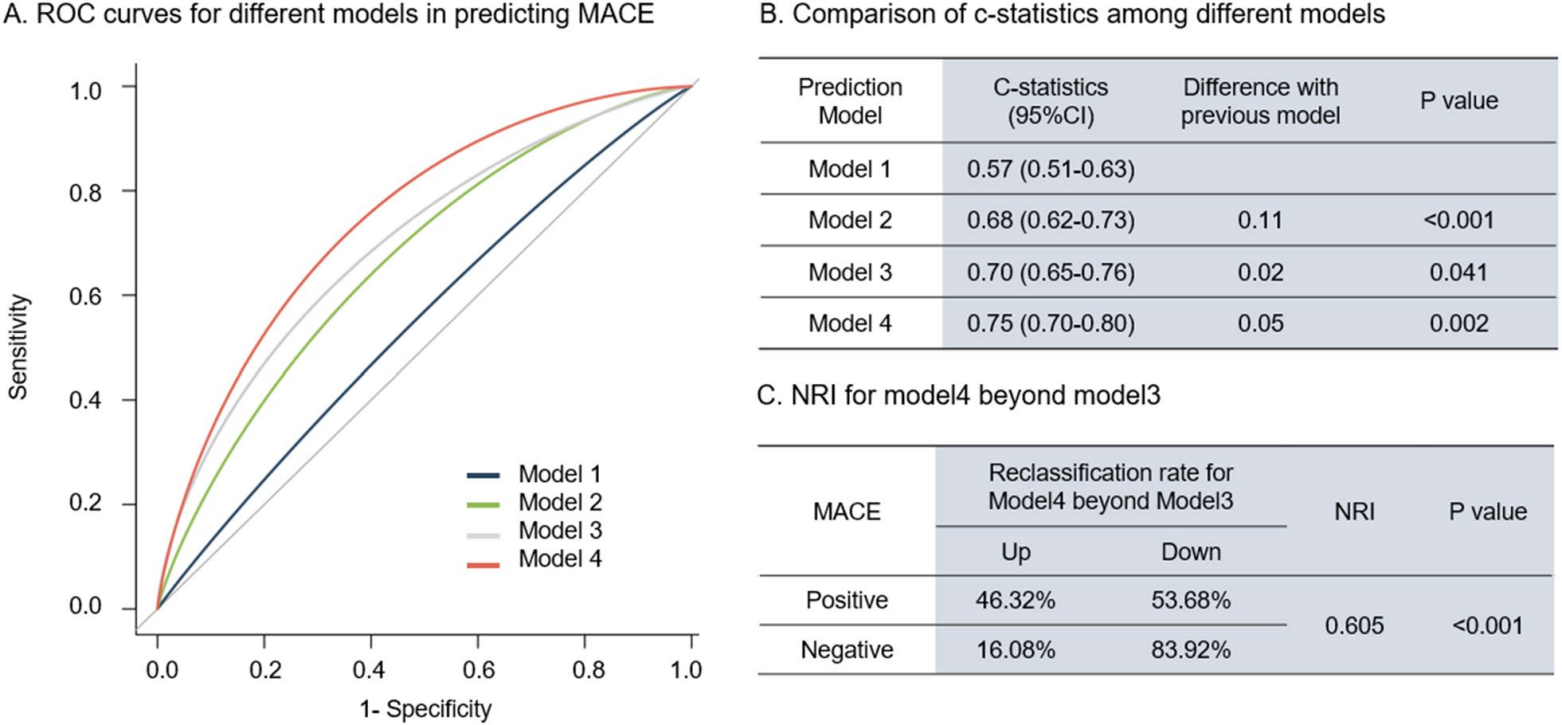
Comparison of discriminant ability of predictive models. To determine incremental discriminatory capacities of Global ΔCT-FFR in addition to clinical variables and atherosclerotic burden as predictor of MACE in diabetic patients with CT-FFR < 0.75, 3 analytic models were constructed as follows: model 1: Framingham risk score; model 2: model 1 + Leiden score; model 3: model 2 + HRP;model4: model3 + Global ΔCT-FFR. *ROC* receiver-operating characteristic, *MACE* major adverse cardiovascular events, *NRI* net reclassification improvement, *HRP* high risk plaque, *GΔCT-FFR* Global ΔCT-FFR (the sum of the trans-lesional CT-FFR gradient in all epicardial vessels)

Of the patients with CT-FFR > 0.75, similar statistical significance of GΔCT-FFR was observed for MACE (HR:2.43, 95% CI 1.45–4.07, *P* < 0.001) (Table 5). Figure 5 shows the C-statistics and discrimination ability of various combination of Framingham risk score, Leiden score, HRP and GΔCT-FFR in diabetic patients with CT-FFR > 0.75. Compared with model 1, discriminant ability was higher of model 2 (C-statistics: 0.68 vs. 0.57; *P* < 0.001), and further promoted by HRP in model 3 (C-statistics: 0.70 vs. 0.68; *P* = 0.04). Combined with GΔCT-FFR, Model 4 showed further increase in discriminant ability (C-statistics: 0.75 vs. 0.70; *P* = 0.002). Net reclassification improvement (NRI) was 0.605 (*P* < 0.001) for model 4 beyond model 3 with the addition of GΔCT-FFR.

**Table 5:** Multivariate analysis of the outcomes in patients with CT-FFR > 0.75

Characteristic	MACE			MACCE		
	Multivariate HR (95% CI)	Wald *x*²	P value	Multivariate HR (95% CI)	Wald *x*²	P value
Age (year)	1.00 (0.98–1.02)	0.039	0.84	1.00 (0.98–1.02)	0.116	0.73
Male	0.87 (0.54–1.40)	0.348	0.56	0.93 (0.61–1.41)	0.116	0.73
Hyperlipidemia	1.83 (1.19–2.81)	7.585	0.006	1.51 (1.04–2.19)	4.772	0.03
Current smoking	1.25 (0.76–2.08)	0.764	0.38	1.20 (0.76–1.87)	0.608	0.44
High-risk plaque	3.62 (2.08–6.30)	20.663	< 0.001	3.18 (1.91–5.31)	19.573	< 0.001
Leiden score	1.71 (1.08–2.71)	5.275	0.02	1.68 (1.13–2.52)	6.442	0.01
GΔCT-FFR ≥ 0.20	2.43 (1.45–4.07)	11.299	0.001	2.11 (1.34–3.34)	10.209	0.001

*CI* confidence interval, *CT-FFR* coronary computed tomography angiography-derived fractional flow reserve, *GΔCT-FFR* global ΔCT-FFR (the sum of the trans-lesional CT-FFR gradient in all epicardial vessels), *HR* hazard ratio, *MACE* major adverse cardiovascular events, *MACCE* major adverse cardiovascular and cerebrovascular events

### Sensitivity analysis

MACE was replaced by MACCE for sensitivity analysis. A similar distribution of event rates was noticed (Fig. 4B and D) (Additional file 1: Table S1), which were 1.61, 5.89 and 9.69 events per 100 person-years for the low-gradient without flow-limiting group (CT-FFR > 0.75 and GΔCT-FFR < 0.20), the high-gradient without flow-limiting group (CT-FFR > 0.75 and GΔCT-FFR ≥ 0.20), and the flow-limiting group (CT-FFR ≤ 0.75) respectively. After adjustment, GΔCT-FFR was still a significant predictor of MACCE (HR: 2.37, 95% CI 1.53–3.66; *P* < 0.001) (Table 4). Of subgroups, an increase was conferred in MACCE risk over those of low-gradient without flow-limiting (the high-gradient without flow-limiting group HR: 2.35, 95% CI 1.52–3.64, *P* < 0.001; the flow-limiting group HR: 3.35, 95% CI 2.04–5.51, *P* < 0.001) (Fig. 4D). Moreover, for patients with CT-FFR > 0.75, GΔCT-FFR was still predictive of MACCE (HR: 2.11, 95% CI 1.34–3.34, *P* = 0.001) (Table 5).

## Discussion

The present study showed that GΔCT-FFR, a novel metric derived from CT-FFR calculation, can act as an independent predictor for clinical outcomes in patients with concomitant diabetes and CAD. Furthermore, for those patients with non-obstructive CAD, GΔCT-FFR could allow for improved risk stratification beyond conventional risk score algorithms. Thus, our findings offered a novel and feasible approach to the risk stratification for a global hemodynamic assessment of coronary artery in diabetic patients.

Previous studies provided insights into the hemodynamic assessment based on CCTA to discriminate the phenotype of physiology and population with high risk and highlighted the importance of CT-FFR in risk stratification. A recent meta-analysis [23] confirmed an inverse association between CT-FFR and MACE, including all-cause mortality or myocardial infarction, among patients with stable CAD during a follow-up of 12 months. A series of trials from the Assessing Diagnostic Value of Non-invasive FFR_CT_ in Coronary Care (ADVANCE) Registry [4–6] and the Analysis of Coronary Blood Flow Using Coronary CT Angiography: Next Steps (NXT) substudy [24] also supported the contribution of CT-FFR to risk identification; a trend toward significantly lower MACE was observed in patients with a negative CT-FFR compared with patients with an abnormal CT-FFR [5]. Moreover, ΔCT-FFR across a specific lesion enhanced the identification of vulnerable plaques that subsequently caused acute coronary syndrome [2], thus helping predict cardiovascular outcomes. Our results aligned with a previous study [25] and extended these observations to diabetic patients with a global coronary atherosclerotic burden. In our cohort, only about 33.7% of the included participants had ≥ 50% stenosis, and approximately 10% had CT-FFR ≤ 0.75, presenting a comparatively lower-risk population. This was probably because of the exclusion of CCTA-derived ICA within 3 months and the inclusion of patients with normal or mild CAD compared with previous studies. The significant increase in the risk for the patients with CT-FFR ≤ 0.75 within 6 months, as shown in Fig. 4, might also be associated with early revascularization in this population. Furthermore, we used 0.75 instead of 0.8 as the cutoff value. Thus, a smaller hemodynamic abnormal population was presented. Considering the increased risk of ischemic stroke and death in diabetic patients compared with MI, the present study broadened the definition of MACE with stroke for sensitivity analysis. In addition, an extended duration of observation was used, resulting in a relatively higher MACE incidence.

In the present study, we further elaborated and presented GΔCT-FFR as the sum of all ΔCT-FFR values related to coronary stenosis. The hypothesis was that GΔCT-FFR might be closely associated with the whole myocardial blood flow. A recent study [26] found that the pathological characteristics of the epicardial coronary artery, including wall shear force and plaque characteristics, can effectively predict the abnormal myocardial blood flow (myocardial blood flow and coronary flow reserve measured by gold standard of PET scan), suggesting that there is a significant correlation between myocardial blood flow and epicardial artery. Another study [27] based on nonobstructive CAD also found a significant correlation between microcirculation endothelial function and epicardial vascular endothelial function, which even occurred in the early stage of the disease. In recent years, hemodynamic metrics have been considered the key mediators in the relationship between epicardial coronary blood flow and myocardial microcirculation blood flow [28, 29]. Notably, GΔCT-FFR included information on the extent of atherosclerosis, which was significantly related to cardiovascular risk [30, 31]. A substudy from the Coronary CT Angiography Evaluation for Clinical Outcomes: An International Multicenter (CONFIRM) registry [30] examined the prognostic value of risk factors and atherosclerotic extents in patients without obstructive CAD. It showed that the latter provided more prognostic information for MACE than the traditional cardiovascular risk factors. A similar conclusion was drawn in a previous study of diabetic patients with nonobstructive CAD [7]. Fournier et al. [25] investigated the value of global FFR and confirmed that the physiological correlate of global atherosclerotic burden was associated with long-term outcomes even in the absence of ischemia-producing stenoses, which was consistent with the present study. However, global FFR was only obtained from a fixed location in the coronary tree and is vulnerable to upstream lesions, whereas our proposed GΔCT-FFR represented a trans-lesional decrease in CT-FFR with a stronger and more direct correlation with ischemia. Of note, in our present study, Leiden score was involved in our final risk model as evaluation for atherosclerosis burden, which is consistent with the previous study [32] that Leiden score would be preferred in DM due to a global anatomic assessment of coronary artery tree.

In addition, the specific pathophysiology supports the applicability of GΔCT-FFR in the diabetic population. Given that diabetic patients are prone to diffuse lesions and microcirculation disorders in coronary artery [33], GΔCT-FFR could represent the decline in blood flow gradient caused by atherosclerosis in the whole coronary artery tree, thus reflecting the overall hemodynamic profile sufficiently and accurately. In particular, GΔCT-FFR could be used as a suitable complementary imaging-derived risk factor for a considerable portion of patients with early-stage diabetes without flow-limiting stenosis. As observed in the present cohort, a comparative higher GΔCT-FFR was found in 18.7% of patients with diabetes having a negative CT-FFR, indicating a population with potentially hemodynamic abnormalities associated with high risk. However, the current guideline [34] fails to address the CT-FFR appropriateness and interpretation recommendation in the presence of diabetes. As suggested recently [20], the results of CT-FFR should always be evaluated in a clinical practice context, taking into account the coronary anatomy and other information. Additional studies are required to address the outcome improvement and appropriateness of GΔCT-FFR in diabetes.

This study had several limitations. First, some inherent characteristics of observational studies due to invalid data, missing information, and survival bias might impact the effect size of target variables. Second, some patients were excluded due to invasive examination within 3 months after CCTA or deferral of CCTA because of patient preference, cost, comorbidities, or other considerations, leading to selection bias. Third, despite a relatively small proportion of patients with severe stenosis, an arbitrary value of CT-FFR was used, which may cause an over- or underestimation of GΔCT-FFR. However, similar value had been rendered in previous studies [22, 35]. Fourth, downstream treatments were not included in the multivariate analysis, which may result in potential confounders. Finally, since the focus of present study is to present the prognostic value of GΔCT-FFR, we did not include the comparison among GΔCT-FFR, ΔCT-FFR, calcium score and quantitative plaque burden, which needs to be further investigated. However, Leiden score was included in our prediction model as a comprehensive evaluation of plaque burden, including stenosis severity, location and plaque composition.

In conclusion, for diabetic patients without flow-limiting coronary stenosis, a greater GΔCT-FFR, which represents a physiological metric of global epicardial coronary flow disturbance, was associated with clinical outcomes at the 5-year follow-up visit. Therefore, our findings offered a novel and feasible approach to improved risk stratification for a global hemodynamic assessment of coronary artery in diabetic patients.

## Supplementary Information

**Additional file 1: Table S1. **Major adverse cardiovascular and cerebrovascular events.

## Data Availability

All data generated or analyzed during this study are included in this published article.

## Abbreviations

CCTA: Coronary computed tomography angiography
CAD: Coronary artery disease
CT-FFR: CCTA - derived fractional flow reserve
ΔCT-FFR: CT-FFR lesional gradient
GΔCT-FFR: The sum of trans-lesional CT-FFR gradient in all epicardial vessels greater than 2 mm
MACE: Major adverse cardiovascular events
MACCE: Major adverse cardiovascular and cerebrovascular events
