# Supplementary material for: Global trans-lesional computed tomography-derived fractional flow reserve gradient is associated with clinical outcomes in diabetic patients with non-obstructive coronary artery disease
Source: Cardiovasc Diabetol. 2023 Jul 26;22:186. doi: 10.1186/s12933-023-01901-9 (PMC10373274; doi:10.1186/s12933-023-01901-9)
Supplement: Supplementary file 1 — Additional file 1: Table S1. Major adverse cardiovascular and cerebrovascular events. [file 12933_2023_1901_MOESM1_ESM.docx]

**Table S1 Major adverse cardiovascular and cerebrovascular events**

| Major adverse cardiovascular and cerebrovascular events | CT-FFR>0.75 （n=1084） | CT-FFR≤0.75 (n=131) | *P* value |
| --- | --- | --- | --- |
| At least one major adverse event | 121(11.2%) | 50(38.2%) | <0.01 |
| Cardiovascular death | 3(0.3%) | 5(3.8%) | <0.01 |
| Nonfatal myocardial infarction | 20(1.8%) | 11(8.4%) | <0.01 |
| Unstable angina requiring hospitalization | 72(6.6%) | 26(19.8%) | <0.01 |
| Stroke | 26(2.4%) | 9(6.9%) | 0.004 |

CT-FFR, coronary computed tomography angiography−derived fractional flow reserve
